# Disparities in health system input between minority and non-minority counties and their effects on maternal mortality in Sichuan province of western China

**DOI:** 10.1186/s12889-017-4765-y

**Published:** 2017-09-29

**Authors:** Yan Ren, Ping Qian, Zhanqi Duan, Ziling Zhao, Jay Pan, Min Yang

**Affiliations:** 1 West China School of Public Health, Sichuan University, Chengdu, 610041 Sichuan People’s Republic of China; 2 Sichuan Provincial Maternal and Child Health Hospital, Chengdu, Sichuan People’s Republic of China; 3 Health and Family Planning Information Centre of Sichuan Province, Chengdu, Sichuan People’s Republic of China; 4 West China Research Center for Rural Health Development, Sichuan University, Chengdu, Sichuan People’s Republic of China; 5 School of Medicine, University of Nottingham, Nottingham, UK

**Keywords:** Ethnic disparity, Maternal mortality rate, Health system, China

## Abstract

**Background:**

The maternal mortality rate (MMR) markedly decreased in China, but there has been a significant imbalance among different geographic regions (east, central and west regions), and the mortality in the western region remains high. This study aims to examine how much disparity in the health system and MMR between ethnic minority and non-minority counties exists in Sichuan province of western China and measures conceivable commitments of the health system determinants of the disparity in MMR.

**Methods:**

The MMR and health system data of 67 minority and 116 non-minority counties were taken from Sichuan provincial official sources. The 2-level Poisson regression model was used to identify health system determinants. A series of nested models with different health system factors were fitted to decide contribution of each factor to the disparity in MMR.

**Results:**

The MMR decreased over the last decade, with the fastest declining rate from 2006 to 2010. The minority counties experienced higher raw MMR in 2002 than non-minority counties (94.4 VS. 58.2), which still remained higher in 2014 (35.7 VS. 14.3), but the disparity of raw MMR between minority and non-minority counties decreased from 36.2 to 21.4. The better socio-economic condition, more health human resources and higher maternal health care services rate were associated with lower MMR. Hospital delivery rate alone explained 74.5% of the difference in MMR between minority and non-minority counties. All health system indicators together explained 97.6% of the ethnic difference in MMR, 59.8% in the change trend, and 66.3% county level variation respectively.

**Conclusions:**

Hospital delivery rate mainly determined disparity in MMR between minority and non-minority counties in Sichuan province. Increasing hospital birth rates among ethnic minority counties may narrow the disparity in MMR by more than two-thirds of the current level.

**Electronic supplementary material:**

The online version of this article (10.1186/s12889-017-4765-y) contains supplementary material, which is available to authorized users.

## Background

Maternal mortality ratio (MMR) is one of the most important indicators reflecting the development of a country’s economy, culture, and healthcare system, and is recognized globally. The fifth Millennium Development Goal 5 (MDG5) intended to improve maternal health, and one of the MDG5 targets is to reduce maternal mortality by 75% in 1990–2015 [1]. According to the World Health Organization (WHO) report, approximately 99% of the global maternal deaths occur in developing countries in 2015 [2]. Reducing maternal mortality is a continuing global priority, particularly in developing countries. The transformative new agenda for maternal health has been laid out as part of the Sustainable Development Goals (SDGs): to reduce the global MMR to less than 70 per 100,000 live births by 2030 (SDG 3.1).

The MMR in China has decreased strikingly from 88.8 maternal deaths per 100,000 live births in 1990 to 21.7 in 2014 [3], and China is one of the few countries achieving Millennium Development Goal 5. The main causes of maternal death were obstetric haemorrhage, hypertensive disorders in pregnancy, heart diseases, amniotic fluid embolism, puerperal infection in 1996, and still were obstetric haemorrhage, hypertensive disorders in pregnancy, heart diseases, amniotic fluid embolism, venous thrombosis and pulmonary embolism in 2014 [4]. Such causes are similar between minority and non-minority population nationwide. Despite the fact that the MMR markedly decreased in China, there has been a significant imbalance among different geographic regions (east, central and west regions). In spite of the fact that China government has made efforts to narrow the gaps in maternal mortality, these gaps persist, and the mortality in the western region remains high. One study found that MMR was fundamentally higher in 2010 in the Western area than in other areas: 46.1 per 100,000 live births, compared with 29.1 for the central region and 17.8 for the east [5]. Other studies on three regions of China also found that west region had higher MMR than the central and east region [6, 7]. According to official statistics from China Health and Family Planning Commission Statistical Yearbook 2015, the MMR in the Sichuan of the Western China is around two circumstances higher than the created districts of east region, for example, Beijing, Tianjin and Shanghai.

Western China only takes up 27% of the population of the nation in 2010 census but accounts for 41% of a total number of maternal deaths according to official statistics from China Health and Family Planning Commission Statistical Yearbook 2011. Western China is notable for its ethnic diversity, 71% of China’s ethnic minority population and 46 different minority groups are registered as living there [8, 9]. Sichuan province is a microcosm of the region as a whole, 6.12% of the ethnic minority population, and 14 different minority groups live in this region according to the 2010 census [10]. Most of minority population in Sichuan live together in counties where their ancestors lived for generations, were difficult to be accessed, where the economic and social development are usually lag behind other counties. To facilitate the development of Sichuan province evenly and to provide special help to the minority population who live in those disadvantaged counties, the government has divided counties in Sichuan province into two categories, minority counties and non-minority counties, based on the number of minority residents, number of townships with minority population, historical factors, economic status and geographic location.

Based on the health systems framework for improving maternal, neonatal and child health outcomes developed by United States Agency for International Development (USAID) in 2011 [11], disparity of maternal deaths reflects inequities of the health systems. Between 2002 and 2014, Sichuan government made substantial investments with supporting policies in the health system, some of these have specifically focused on the minority areas in the province. It has critical ramifications to know whether the government endeavours had progressed health system measures and narrowed disparities among minority and non-minority counties, which improvement may impact on reduction or change trend in MMR and explain disparities between minority and non-minority counties, as well as the county level variance. The evidence from China on the relationship between health systems and MMR can be found in some studies, such as higher hospital beds per 1000 population, higher length of highways, higher GDP, higher hospital delivery rate, higher utilization of prenatal care, more village doctors and lower illiteracy rate are persistently associated with lower MMR [12–16]. However, few study analyzed what we need to know.

While various publications in English had particularly centred around maternal mortality in China, yet just a single study reported the effect of family planning on maternal mortality in Sichuan from 1989 to 1991 [17]. In this study, we used county-level data from Sichuan Province for the years of 2002, 2006, 2010, 2014 to describe the socio-economic environment, maternal health policies and programmes, health human resources, health infrastructure, maternal health care services and their explanation power in reducing ethnic disparities in MMR. This findings may help policy-makers to make well-informed decisions to optimize health system investments,which eventually could effectively reduce regional disparity in MMR.

## Methods

### Data and variables

The MMR was expressed as the number of maternal deaths per 100,000 live births within one year. Maternal deaths are defined as deaths of women who are “pregnant or within 42 days of termination of pregnancy, regardless of the span and the site of the pregnancy, from any cause related or irritated by the pregnancy or its management, but not from accidental or incidental causes” [18]. The maternal death and live births data of 67 minority and 116 non-minority counties for the years of 2002, 2006, 2010 and 2014 were provided by the Sichuan Maternal and Child Health Hospital to where all health facilities in the province report health outcome data and health system data annually as required by the central government. The data forms a two-level hierarchical structure with repeated maternal deaths nested within the county.

We reviewed national and provincial policies related to MMR, and applied a USAID health systems framework for improving maternal, neonatal and child health [9]. The health systems aspects that might be associated with the county MMR were divided into the following groups: (1) Social environment was measured by GDP per capita, rate of urbanization and highway per land, the average travelling time to the nearest hospital (ATH); (2) Health human resources ware measured by the number of registered doctors per 1000 population, the number of registered nurses per 1000 population, the service areas of registered doctors, the service areas of registered nurses, the density of registered doctors; (3) Health infrastructure was measured by the number of hospital beds per 1000 population, the number of health institutes (all facilities which provide health services, such as county hospitals, township hospitals, community health centers, village clinics) per 1000 population and the number of hospitals per 100,000 population; (4) Maternal health care service was measured by the proportion of pregnant women who delivered in hospitals, received one or more antenatal visits, received postnatal care and received systematic care.

The maternal health care service data came from Sichuan Maternal and Child Health Hospital, the health infrastructure and health human resources data came from Sichuan Health Statistic Information Centre, and the social environment data came from the Sichuan Statistical Year Book.

### Statistical analysis

We firstly described socio-economic and demographic of minority and non-minority counties of Sichuan province and then documented variation in relevant policies and programmes for maternal health. We used mean, standard deviation and annual change rate to describe the region variation in health human resources, health infrastructure and maternal health care service between 2002 and 2014. We briefly portrayed the considerable changes that have occurred in the uptake of maternal mortality in each region over the same period and explored reasons for maternal mortality reduction using a two-level Poisson regression analysis.

Based on the number of maternal deaths and live births in each county over four-time points in the years of 2002, 2006, 2010, 2014, some two-level Poisson models with counties at level 2 and times at level 1 was constructed to estimate the difference in MMR between minority and non-minority counties under different conditions. The Model 1 estimated the ethnic distinction without considering any health system indicators. Then to access impacts of health systems on such difference, Models 2 to 7 adjusted for one health system covariate X individually in each model. At last, all health system covariates were considered in Model 8. The percentages of changes in the parameter estimates between Model 1 and Model 2 to 8 were calculated to measure contributions of health system indicators.

We used package SAS 9.3 [19] for descriptive analysis and MLwiN 2.30 [20] for all modelling analysis.

## Results

### Socio-economic and demographic of Sichuan province

**Fig. 1:**
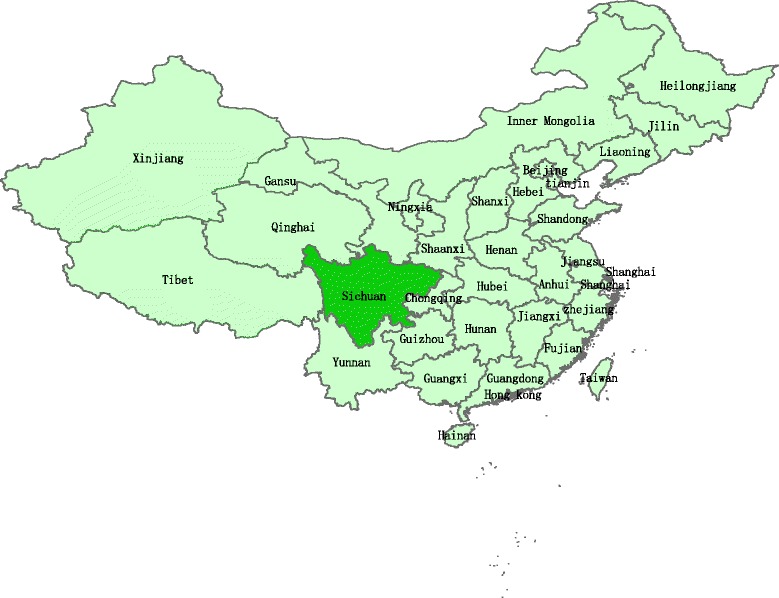
The area of Sichuan province in China map

**Fig. 2:**
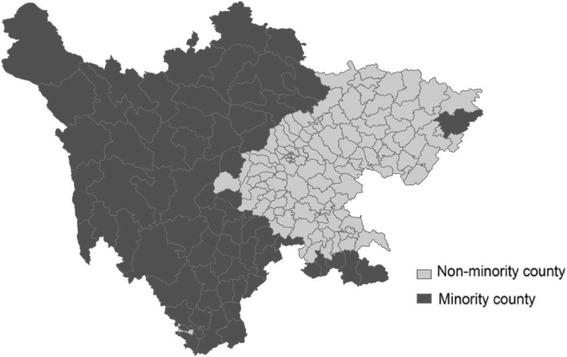
Distribution of minority and non-minority counties in Sichuan province by Sichuan Statistical Yearbook 2015

Sichuan province is dark green area in China map (Fig. 1), including 67 minority and 116 non-minority counties (Additional file 1: Table S1). Minority counties are mostly located on the mountain or hilly areas to the west of Sichuan as shown in Fig. 2 and have the concentrated population of ethnic minorities. The socio-economic and demographic characteristics of the two regions in Table 1 suggest that minority area is larger, has a lower GDP per capita, less urbanisation rate, fewer highway per land and much sparsely populated than the non-minority area in 2014. The average travelling time to the nearest hospital (ATH) was calculated per a shortest-path analysis method [21]. The ATH of minority counties in 2012 with a mean at 84.5 min and standard deviation (SD) at 33.6 was significant disadvantages (*p* < 0.001) than that of non-minority counties (mean = 42.6, SD = 18.5).

**Table 1:** General information of socio-economic and demographics of Sichuan province in 2014

District	Minority counties	Non-minority counties
No. of County(%)	67(36.6)	116(63.4)
Land size (km^2^)(%)	344,551(69.9)	148,231(30.1)
Population (10,000 persons)(%)	1388.1(15.1)	7775.5(84.9)
GDP per capita(yuan)	24,137	32,515
Rate of urbanization(%)	15.9	31.7
Highway per land(km)	0.22	1.41

### Relevant policies and programs for maternal health

Between 2002 and 2014, central and Sichuan government designed some policies and invested substantial funds in the reduction of maternal mortality (Additional file 2: Table S2). A few methods gave financial subsidies to support rural women to give birth in the hospital, such as “reducing maternal mortality and eliminating neonatal tetanus” and “Hospital Delivery Subsidy Project in Rural Areas”. Some programs specifically focused on maternal and child health of the minority areas in Sichuan, such as “ Improving Hospital Delivery Rate in 31 Minority Counties”, “Supporting the maternal and child health Counterparts in Minorities-inhabited Regions”, “ten-year action plan of health industry development in minorities-inhabited regions” and “1,000 Health Cadres Supporting Minorities-inhabited Regions”. These programs aimed to improve the hospital delivery and maternal and child health service capacity in minority counties. Through “Public health institutions standardisation construction” project, more health institutions constructed nationwide. The New Rural Cooperative Medical Scheme (NCMS) additionally went for empowering rural hospital delivery. Pregnant women participate in NCMS get appropriate reimbursement with the different rate for caesarian and vaginal deliveries in various level hospitals. Some programs might not directly reduce the maternal mortality rate, but could improve the maternal and child health, such as “Mother-baby HIV, hepatitis B and syphilis translation examination” to cover the whole province, “ Cervical and breast cancer screening to cover eligible rural women” and “ Giving the rural women who prepare become pregnant free folic acid supplementation”.

### Health human resources

Statistics in Table 2 show substantial variation between minority and non-minority areas in human health resources during the last decade. The number of registered doctors per 1000 population and density of registered doctors per square kilometre increased over time among non-minority counties but decreased over time among minority counties. As a result service areas per registered doctors increased considerably in minority counties and reduced in non-minority counties. The quantity of registered nurses per 1000 population was expanded with various change rates after some time in non-minority counties and minority counties, hence the service areas of registered nurses reduced in all counties. Overall, minority counties had reduced registered doctors and increased service areas per registered doctor, compared to non-minority counties.

**Table 2:** The mean and standard deviation (SD) of health human resources indicators by ethnic groups

Indicators	Minority counties, *N* = 1388.1 (10,000 persons)			Non-minority counties, *N* = 7775.5 (10,000 persons)		
	2002 Mean(SD)	2014 Mean(SD)	Annual change rate(%)	2002 Mean(SD)	2014 Mean(SD)	Annual change rate(%)
No of registered doctors (per 1000 pop)	1.94(0.96)	1.53(0.89)	−1.96	1.6(1.4)	2.2(1.5)	2.69
No of registered nurses (per 1000 pop)	0.73(0.39)	1.34(0.87)	5.19	0.92(1.1)	2.2(2.1)	7.54
Density of registered doctors	0.34(0.20)	0.33(0.26)	−0.25	1.9(4.0)	2.5(4.7)	2.31
Density of registered nurses	0.14(0.12)	0.31(0.31)	6.85	1.2(2.8)	2.7(6.0)	6.99
Area per doctor serve (km^2^)	38.5(51.0)	51.4(74.9)	2.44	2.1(2.1)	1.5(1.5)	−2.76
Area per nurse serve (km^2^)	171.2(512.4)	66.4(104.1)	−7.59	5.6(6.5)	2.0(2.4)	−8.22

### Health infrastructure

Health infrastructure indicators in Table 3 exhibit distinctive change designs between ethnic counties. The number of hospital beds per 1000 population increased faster over time among non-minority counties than minority counties, doubled from 2.7 in 2002 to 5.6 in 2014. The number of health institutes per 1000 population increased and the number of hospitals per 100,000 population somewhat lessened after some time among minority counties, while as the two indicators were the different way over time among non-minority counties. Since the health institutes include township hospitals, community health center(station) and village clinics, the increase of health institutes in number may suggest some effects of recent government policy in strengthening preventive care, such as “Public health institutions standardization construction” project, in 2009-2014, government invested 1.599 billion Chinese Yuan(CNY) for the standardization construction of 2089 township hospitals, 151 community health center (station), 13,748 village clinics. These health institutions mainly provide preventive health care and some basic curative care. Results recommended a general lessened hospital care but increased preventive care facilities in minority counties during the last decade.

**Table 3:** The mean and standard deviation (SD) of health infrastructure indicators by ethnic groups

Indicators	Minority counties			Non-minority counties		
	2002 Mean(SD)	2014 Mean(SD)	Annual change rate(%)	2002 Mean(SD)	2014 Mean(SD)	Annual change rate(%)
No of hospitals (per 100,000 pop)	3.1(2.2)	2.8(1.7)	−0.84	1.7(1.6)	2.2(1.6)	2.17
Service area per hospital (km^2^)	2418(2318)	2146(2167)	−0.99	302(293)	188(247)	−3.87
Hospital beds (per 1000 pop)	2.7(1.2)	4.2(2.0)	3.75	2.7(2.1)	5.6(3.5)	6.27
No of health institute (per 1000 pop)	1.3(0.5)	1.7(0.9)	2.26	0.86(0.25)	0.85(0.23)	−0.10
Service area per health institute (km^2^)	64(86)	35(44)	−4.91	2.8(2.9)	2.7(2.4)	−0.30

### Maternal health care service

The maternal health care service indicators in Table 4 suggest that all indicators among minority counties showed greater improvement than those of non-minority counties during the last decade. However, in 2014 the four indicators were still notably lower among minority counties than non-minority counties with much greater variability among minority counties. It suggests that minority counties still have much potential to improve the maternal health care service.

**Table 4:** The mean and standard deviation (SD) of maternal health care service indicators by ethnic groups

Indicators	Minority counties			Non-minority counties		
	2002 μ(SD)	2014 μ(SD)	Annual change rate(%)	2002 μ(SD)	2014 μ(SD)	Annual change rate(%)
Hospital delivery rate(%)	38.0(21.2)	86.0(13.2)	7.0	78.0(17.8)	99.9(0.1)	2.1
Prenatal care rate(%)	67.6(23.6)	86.4(13.6)	2.1	91.8(7.3)	98.2(1.5)	0.6
Postnatal care rate(%)	61.5(25.1)	84.5(15.4)	2.7	89.9(8.2)	97.6(1.6)	0.7
Systematic care rate(%)	46.4(27.8)	79.6(17.0)	4.6	78.0(17.8)	96.3(2.1)	1.8

Prenatal care rate: the percentage of pregnant women received one or more number of antenatal examinations account for the live birth. Postnatal care rate: the percentage of pregnant women after delivery received one or more number of postnatal visit within 28 days account for the live birth. Systematic care rate: the percentage of pregnant women from pregnancy to delivery 28 days received early pregnancy antenatal examination, at least one antenatal examinations, new method delivery and postnatal visit account for the live birth

### Difference in change trends in MMR

**Fig. 3:**
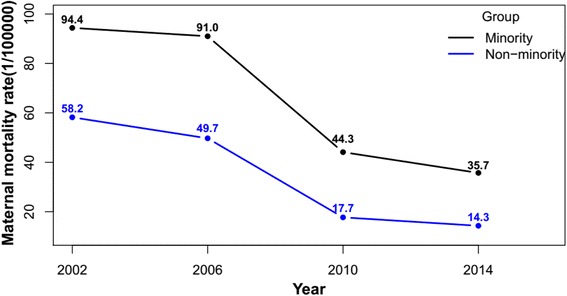
Raw maternal mortality rate (1/100,000) by year and ethnic group

The MMR decreased over the last decade as shown in Fig. 2, with the fastest declining rate from 2006 to 2010. The minority counties had higher raw MMR in 2002 than non-minority counties (94.4 VS 58.2), and remaining higher in 2014 (35.7VS 14.3), but with lower annual reduction rate (7.8% VS 11.0%). The difference of raw MMR between minority and non-minority counties decreased from 36.2 to 21.4. Model estimates in Table 5 confirmed that the time for change parameters was significantly negative in all models, which indicated over time reducing of adjusted MMR regardless what covariates were adjusted for (Fig. 3).

**Table 5:** Estimates of effects of health system indicators on MMR by 2-level Poisson models

Indicators	Model 1 *β(SE)*	Model 2 *β(SE)*	Model 3 *β(SE)*	Model 4 *β(SE)*	Model 5 *β(SE)*	Model 6 *β(SE)*	Model 7 *β(SE)*	Model 8 *β(SE)*
Time for change	−0.112 (0.008)**	−0.062 (0.010)**	−0.101 (0.008)**	−0.109 (0.008)**	−0.112 (0.007)**	−0.036 (0.014)*	−0.112 (0.001)*	−0.045 (0.015)**
Minority	0.780 (0.103)**	0.199 (0.118)	0.431 (0.114)**	0.665 (0.103)**	0.582 (0.108)**	0.614 (0.097)**	0.592 (0.119)	0.019 (0.121)
County variance	0.199 (0.042)**	0.105 (0.030)**	0.135 (0.034)**	0.169 (0.039)**	0.159 (0.037)**	0.134 (0.034)**	0.178 (0.040)*	0.067 (0.025)*
RHD		−0.017 (0.002)**						−0.008 (0.003)*
PCR			−0.014 (0.002)**					−0.007 (0.003)*
DRD				−0.096 (0.028)**				−0.050 (0.025)*
NHI					0.417 (0.098)**			0.315 (0.097)**
PGDP						−0.486 (0.079)**		−0.227 (0.091)*
ATH							0.056 (0.002)**	0.001 (0.002)
Change rate explained %		44.6	9.8	2.7	0.0	67.9	0.0	59.8
Ethnic different explained %		74.5	44.7	14.7	25.4	21.3	24.1	97.6
County variance explained %		47.2	32.2	15.1	20.1	32.7	10.6	66.3

RHD: percentage of pregnant women who delivered in hospitals; PCR: prenatal care rate; DRD: number of registered (including assistant) doctors per 1000 population per square km; NHI: number of health institutes per 1000 population; PGDP: the ln(GDP per capita); ATH: the average travelling time to the nearest hospital. *P < 0.05, **P < 0.001

### Effects of health systems on MMR

The results of Model 8 in Table 5 indicate five health system factors that were associated with the reduction in MMR. They were increased hospital delivery rate (RHD) (*P* = 0.006), increased prenatal care rate (PCR)(*P* = 0.022), increased registered (including assistant) doctors per 1000 population per square km (DRD) (*P* = 0.048), decreased health institutes per 1000 population (NHI) (*P* = 0.001) and increased GDP per capita (PGDP) (*P* = 0.013).

The percentage of ethnic group disparity, the change trend and county variance explained by health system indicators is presented in the last three rows of Table 5. It shows that the hospital delivery rate (RHD) alone explained 74.5% of the difference in MMR between minority and non-minority counties and 47.2% of the county variance respectively. The GDP per capita (PGDP) alone explained the most MMR decreasing by 67.9%. From Model 8 we can see that all system indicators together explained 97.6% difference in MMR between ethnic groups, 59.8% decreasing of MMR over time and 66.3% county variance.

## Discussion

The study showed that Sichuan government had invested substantial funds in some specific programs to target on the improvement of maternal and child health of the minority areas since 2000. The disparities in MMR and health system between minority and non-minority counties in Sichuan province narrowed over time. But the health human resources, health infrastructure, and maternal health care service in minority counties were still lacked behind of non-minority counties, in addition to a lower social and economic environment than the latter. The study likewise shows that better economic environment, more health human resources and more maternal health care services were essentially connected with reduced MMR. All six health system factors together explained 97.6% ethnic group disparity, with most accountable factors being the hospital delivery and prenatal care. The health system factors together attributed 66.3% county variation, about one-third of county level variation has remained unexplained. The GDP per capita (PGDP) alone explained the 67.9% MMR decreasing.

The fastest declining rate in MMR during 2006 to 2010 could be due to the impact of policies. During this period, health authority in Sichuan province invested a large number of funds in improving hospital delivery, including programme of “Reducing Maternal Mortality and Eliminating Neonatal Tetanus (RMMENT)” from 2000 to 2009, of “Hospital Delivery Subsidy Project in Rural Area (HDSPRA)” started in 2009, of “New Rural Cooperative Medical Scheme (NCMS)” started in 2007. One study found that the RMMENT program has altogether expanded the hospital delivery rate and essentially diminished the MMR in mid-western China [22].

Ethnic minority counties had a lower level in social and economic environment indicators than non-minority counties. The increased per GDP was associated with decreased MMR and explained the most MMR declining trend. Other provinces of China studies also found this association [13, 23]. Economic environment was also significantly associated with other low and middle-income countries, but little related to developed countries [24]. The impact of economic status on MMR may be through other factors such as maternal education, expenditure on health care, quality of care services. Many minority counties are the least developed counties located in mountainous areas. The GDP per capita of minority counties was persistently lower than that of non-minority counties in Sichuan over time.

Increased number of qualified doctors was partly attributable to reduce MMR disparity, has been reported by some previous studies [25–27], which is consistent with this study. We also found a decreasing trend in qualified doctors among minority counties from 2002 to 2014, in contrast to an increasing trend among non-minority counties. This finding suggested an increasing gap in qualified doctors between minority and non-minority countries, which could cause the ethnic disparity in MMR. Further research in this area is guaranteed.

We found an increased number of health institutes per 1000 population and decreased hospital number among minority counties in Sichuan province and a positive relationship between the institution indicator and MMR, i.e. more health institutions and higher MMR. These findings may suggest some effects of recent government policy in strengthening preventive care, such as “Public health institutions standardisation construction” project. During 2009-2014, the government invested 1.599 billion Chinese Yuan (CNY) for the standardisation construction of 2089 township hospitals, 151 community health centre (station), 13,748 village clinics. Health institutes included township hospitals, community health centre (station) and village clinics. However, most of those health care facilities did not provide curative care nor delivery services. Instead, they collected and managed medical information by request of the health authority. Thus, more maternal deaths may be reported where more health care institutions were in real life. A study by Zanini, RR. et al. also found that increased number of hospitals per 100 thousand inhabitants was associated with a decreased infant mortality rate [28]. No study estimated the association between health institutes and MMR.

The maternal health care services markedly improved over time among all counties, with the faster annual change rate of minority counties than non-minority counties. As a key finding that higher level of hospital delivery rate and antenatal care rate were associated with a lower level MMR, the previous alone clarified the most contrast in MMR between minority and non-minority counties and county variance. Increased hospital delivery rate being associated with decreased MMR has been evidenced by other studies in China [13, 16]. Hospital delivery with skilled attendants may decrease the risk of maternal death. Health authority in Sichuan province invested a large number of funds in improving hospital delivery, including programme of “Reducing Maternal Mortality and Eliminating Neonatal Tetanus” from 2000 to 2009 (RMMENT), of “Hospital Delivery Subsidy Project in Rural Area” started in 2009 (HDSPRA), and of “Improving Hospital Delivery Rate in 31 Minority Counties” started in 2010 (IHDRMC). One study found that the RMMENT program has fundamentally expanded the hospital delivery rate and essentially decreased the MMR in mid-western China [22]. The IHDRMC project has increased the hospital delivery rate in these minority counties from 30.1% in 2010 to 71.0% in 2014 [29]. The New Rural Cooperative Medical Scheme (NCMS) started in 2007 in Sichuan had reimbursement in favour of ethnic minority areas with different rate for caesarean and vaginal deliveries in different level hospitals [30], the NCMS has resulted in increased use of health services and hospital delivery services [31]. But there are still 29% of pregnant women in 31 Minority Counties who refuse to deliver in the hospitals according to our recent unpublished qualitative study. This is not only an economic issue but also issues of traditional culture and beliefs [15]. It is vital to comprehend what cultural and belief factors may impact the utilisation of health services in minority counties so that culturally appropriate health education programs can be run efficiently to promote hospital delivery. We are currently extending our study to identify minority case counties that had the highest and lowest RHD with low or high MMR to develop specific education and treatment program for targeted intervention.

The study has some limitations. First, the maternal death data was collected from the health administrative system, which might have under-reporting bias due to some target setting pressure. So the MMR used in this study could be different from studies where bias adjustment was made. Such bias could be greater in minority counties than non-minority counties in early period because of their much higher MMR, and then reduced more following the improvement of health systems over time as indicated in this study. This suggests that the real gap in the MMR and in the time trends between the two groups could be fractural rather than constant, and the attribution of health system factors to the differences could be variable in percentages accordingly. So the results of this study should be interpreted as descriptive and not as predictive. Further research based on individual data is required on this account. Second, we cannot acquire the individual level data, so the association between the MMR and health system factors cannot be interpreted at the individual level. In spite of these constraints, the study gives vital findings that health system factors and their correlation with reduction of MMR disparity in the past decade.

In summary, this study reviewed maternal health policies and programs on MMR, presented differences between minority and non-minority counties in the MMR and the socio-economic environment, health human resources, health infrastructure, maternal health care services in Sichuan province, and examined explicitly impact of health system indicators on the MMR disparity related to ethnicity. The strong pieces of evidence on ethnic minority related disparity in MMR and on gaps in health system development indicate that efforts to lower MMR in Sichuan province ought to keep on focusing on minority areas later on. Improving hospital delivery and rising economic level of minority areas are still key measures to act on. The finding may reach out to other provinces in western China due to similar geographic, demographic and socio-economic environment [32].

## Conclusions

The MMR decreased over the last decade, with the fastest declining rate from 2006 to 2010. The disparity of raw MMR between minority and non-minority counties decreased. The better socio-economic condition, more health human resources and higher maternal health care services rate were associated with lower MMR. Hospital delivery rate mainly determined disparity in MMR between minority and non-minority counties in Sichuan province. The economic mainly determined the change rate of MMR. The results of the present study can help health authorities to understand that the hospital delivery rate is the key healthcare system determinants for the ethnic minority-related disparity in MMR, and rising economic level is key measures to decrease the MMR.

## Additional files

Additional file 1: Table S1. List of the minority and non-minority counties/districts in Sichuan province. (DOCX 13 kb)

Additional file 2: Table S2. Important policies or events to improve maternal health in Sichuan province, 2002-2014. (DOCX 16 kb)

## Abbreviations

ATH: Average travelling time to the nearest hospital
DRD: Registered (including assistant) doctors per 1000 population per square km
GDP: Gross domestic product
HDSPRA: Hospital Delivery Subsidy Project in Rural Area
IHDRMC: Improving Hospital Delivery Rate in 31 Minority Counties
MMR: Maternal mortality rate
NCMS: New Rural Cooperative Medical Scheme
NHI: Health institutes per 1000 population
PCR: Prenatal care rate
PGDP: GDP per capita
RHD: Hospital delivery rate
RMMENT: Reducing Maternal Mortality and Eliminating Neonatal Tetanus
SAS: Statistics Analysis System
SD: Standard deviation
USAID: United States Agency for International Development
WHO: World Health Organization
