# Supplementary material for: Disparities in health system input between minority and non-minority counties and their effects on maternal mortality in Sichuan province of western China
Source: BMC Public Health. 2017 Sep 29;17:750. doi: 10.1186/s12889-017-4765-y (PMC5622414; doi:10.1186/s12889-017-4765-y)
Supplement: Supplementary file 1 — List of the minority and non-minority counties/districts in Sichuan province. (DOCX 13 kb) [file 12889_2017_4765_MOESM1_ESM.docx]

Additional file 1: Table S1 List of the minority and non-minority counties/districts in Sichuan province

| **Minority counties/districts(N=67)** | **Non-minority counties/districts(N=116)** |
| --- | --- |
| Renhe, Miyi, Yanbian, Xuyong, Gulin, Beichuan, Pingwu, Jinkouhe, Ebian, Mabian, Yingjing, Baoxing, Hanyuan, Shimian, Xuanhan, Gongxian, Junlian, Xingwen, Pingshan, Wenchuan, Lixian, Maoxian, Songpan, Jiuzhaigou, Jinchuan, Xiaojin, Heishui, Maerkang, Rangtang, Abaxian, Ruoergai, Hongyuan, Kangding, Luding, Danba, Jiulong, Yajiang, Daofu, Luhuo, Ganzixian, Xinlong, Dege, Baiyu, Shiqu, Seda, Litang, Batang, Xiangcheng, Daocheng, Derong, Xichang, Muli, Yanyuan, Dechang, Huili, Huidong, Ningnan, Puge, Buto, Jinyang, Zhaojue, Xide, Mianning, Yuexi, Ganluo, Meigu, Leibo | Jinjiang, Qingyang, Jinniu, Wuhou, Chenghua, Longquanyi, Qingbaijiang, Xindu, Wenjiang, Jintang, Shuangliu, Pixian, Dayi, Pujiang, Xinjin, Dujiangyan, Pengzhou, Qionglai, Chongzhou, Ziliujing, Gongjing, Daan, Yantan, Rongxian, Fushun, East District, West District, Jiangyang, Nanxi, Longmatan, Luxian, Hejiang, Jinyang, Zhongjiang, Luojiang, Guanghan, Shifang, Mianzhu, Hucheng, Youxian, Santai, Yanting, Anxian, Zitong, Jiangyou, Lizhou, Zhaohua, Chaotian, Wangcang, Qingchuan, Jiange, Cangxi, Chuanshan, Anju, Pengxi, Shehong, Daying, Neijiang Downtown, Dongxing, Weiyuan, Zizhong, Longchang, Leshan Downtown, Shawan, Wutongqiao, Qianwei, Jingyan, Jiajiang, Muchuan, Emeishan, Shunqing, Gaoping, Jialing, Nanbu, Yingshan, Pengan, Yilong, Xichong, Langzhong, Dongpo, Renshou, Pengshan, Hongya, Danling, Qingshen, Cuiping, Nanxi, Yibin, Jiangan, Changning, Gaoxian, Guanganqu, Qianfeng, Yuechi, Wusheng, Linshui, Huaying, Tongchuan, Dachuan, Kaijiang, Dazhu, Quxian, Wanyuan, Yucheng, Mingshan, Tianquan, Lushan, Bazhou, Enyang, Tongjiang, Nanjiang, Pingchang, Yanjiang, Anyue, Lezhi, Jianyang |
