# Supplementary material for: Disparities in health system input between minority and non-minority counties and their effects on maternal mortality in Sichuan province of western China
Source: BMC Public Health. 2017 Sep 29;17:750. doi: 10.1186/s12889-017-4765-y (PMC5622414; doi:10.1186/s12889-017-4765-y)
Supplement: Supplementary file 2 — Important policies or events to improve maternal health in Sichuan province, 2002-2014. (DOCX 16 kb) [file 12889_2017_4765_MOESM2_ESM.docx]

Additional file 2: Table S2 Important policies or events to improve maternal health in Sichuan province, 2002-2014.

| **Time** | **Important policies or events** | **content summary** |
| --- | --- | --- |
| 2000 | Reducing maternal mortality and eliminating neonatal tetanus | This programme was implemented from 2000 to 2009, with a focus on increasing facility births. The programme was implemented in 52 counties in 2000, expanding coverage to all counties in 2009. |
| 2007 | The New Rural Cooperative Medical Scheme (NCMS) | NCMS was introduced in 2003 in China and started in 2007 in Sichuan. To encourage rural hospital delivery, pregnant women participate in NCMS get appropriate reimbursement with different rate for caesarean and vaginal deliveries in different level hospitals. |
| 2009 | Hospital Delivery Subsidy Project in Rural Area | This program pays 500 Chinese Yuan(CNY) for each rural pregnant woman who had hospital delivery. In 2009-2014, the accumulative subsidy fund was 1.455 billion CNY. |
| 2009 | Giving the rural women who prepare become pregnant free folic acid supplementation | In 2010-2014, the central and provincial government accumulatively invested 70.76 million CNY. In 2014, the taking folic acid rate was 95.75%. |
| 2009 | Cervical and breast cancer screening to cover eligible rural women | In 2009-2014, the central government accumulatively invested 94.86 million CNY, the provincial invested 64.696 million CNY for the "two" cancer screening. |
| 2009 | Public health institutions standardization construction | In 2009-2014, government invested 1.599 billion CNY for the standardization construction of 2089 township hospitals, 151 community health center (station), 13748 village clinics. |
| 2010 | Five antenatal visits free of charge | The content of antenatal care included ultrasound, HIV, syphilis and hepatitis B testing. The provincial government invested 270 million CNY for equipment to improve the maternal health service. |
| 2010 | Improving Hospital Delivery Rate in 31 Minority Counties | The project covered for establishing pre-delivery spots, supporting and conducting trainings of midwifery technique, reimbursement of transportation cost for hospitalized delivery, and pays 100 dollars CNY to each pregnant woman in the project counties who had hospital delivery. The project has increased the hospital delivery rate in these minority counties from 30.1% in 2010 to 71.0% in 2014. |
| 2010 | Mother-baby HIV, hepatitis B and syphilis translation examination to cover the whole province. | In 2010-2014, the central and provincial government accumulatively invested 94.86 million CNY, the provincial invested 477 million CNY for these examinations. In 2014, the provincial mother-baby HIV, hepatitis B and syphilis translation examination rate was 98.46%, 98.49% and 98.46%, respectively. |
| 2010 | Supporting the maternal and child health Counterparts in Minorities-inhabited Regions | Establishing long-term stable support between:  52Grade 3A hospitals and 141 autonomous prefecture and county hospitals in minorities-inhabited regions; 92Grade 2A hospitals and 294 township hospitals in minorities-inhabited regions; 70 maternal and child health hospitals and 70 maternal and child health hospitals in minorities-inhabited regions. |
| 2011 | ten-year action plan of health industry development in minorities-inhabited regions | In 2011, Sichuan launched the Plan, and invested 62 million CNY which belongs to the Maternal and child health special funds in minorities-inhabited regions to improve maternal and child health service capacity in minority counties. |
| 2012 | 1,000 Health Cadres Supporting Minorities-inhabited Regions | From 2012 to 2020, 1,000 health managerial cadres selected from province-level health administration, provincial and central medical care institutes in Sichuan and 18 inland cities will be sent to temporarily work in health administration and medical care institutes in minorities-inhabited regions. |
